# Protein Degradation by E3 Ubiquitin Ligases in Cancer Stem Cells

**DOI:** 10.3390/cancers14040990

**Published:** 2022-02-16

**Authors:** Macarena Quiroga, Andrea Rodríguez-Alonso, Gloria Alfonsín, Juan José Escuder Rodríguez, Sara M. Breijo, Venancio Chantada, Angélica Figueroa

**Affiliations:** Epithelial Plasticity and Metastasis Group, Instituto de Investigación Biomédica de A Coruña (INIBIC), Complexo Hospitalario Universitario de A Coruña (CHUAC), Sergas, Universidade da Coruña (UDC), 15006 A Coruña, Spain; Macarena.Quiroga.Fernandez@sergas.es (M.Q.); andrea.rodriguez.alonso@sergas.es (A.R.-A.); maria.gloria.alfonsin.rey@sergas.es (G.A.); juan.jose.escuder.rodriguez@serga.es (J.J.E.R.); Sara.Martinez.breijo@sergas.es (S.M.B.); Venancio.chantada.abal@sergas.es (V.C.)

**Keywords:** cancer stem cells, post-translational modification, protein degradation, ubiquitination, E3 ubiquitin ligases

## Abstract

**Simple Summary:**

The aim of this review was to discuss the fundamental role of E3 ubiquitin ligases in controlling cancer stem cells. It will be surmised that protein degradation controlled by the E3 ubiquitin ligases plays a fundamental role in the self-renewal, maintenance and differentiation of cancer stem cells, highlighting its potential as an effective therapeutic target for anticancer drug development.

**Abstract:**

Cancer stem cells are a small subpopulation within the tumor with high capacity for self-renewal, differentiation and reconstitution of tumor heterogeneity. Cancer stem cells are major contributors of tumor initiation, metastasis and therapy resistance in cancer. Emerging evidence indicates that ubiquitination-mediated post-translational modification plays a fundamental role in the maintenance of cancer stem cell characteristics. In this review, we will discuss how protein degradation controlled by the E3 ubiquitin ligases plays a fundamental role in the self-renewal, maintenance and differentiation of cancer stem cells, highlighting the possibility to develop novel therapeutic strategies against E3 ubiquitin ligases targeting CSCs to fight cancer.

## 1. Introduction

Human cancers are heterogeneous diseases containing different subsets of cells [1]. Within the heterogeneity, it is becoming increasingly apparent that all human cancers harbor a subpopulation of cancer stem cells (CSCs) with tumor initiating capacity compared to the bulk tumor cell population. CSCs are characterized by self-renewing capabilities and the potential to differentiate into cells that comprise the tumor (multi-potency) [2]. Many publications have reported the identification of CSCs in several types of tumors including breast cancer, leukemia, brain cancer and colorectal cancer [3,4,5,6,7]. Although genomic instability and epigenetics play a crucial role in CSC function, in recent years, other levels of regulation, including translational and post-translational control, have emerged as fundamental regulators [8,9]. Ubiquitination is a post-translational modification that consists of the labeling of substrate proteins with a molecule of ubiquitin (Ub) inducing its degradation. As a consequence, this process controls the “quantity” and “quality” of specific proteins, ensuring cell homeostasis. Ubiquitination plays a fundamental role in the maintenance of CSCs [10]. In the ubiquitination process, the E3 Ub ligases are key components of the enzymatic reaction as they are responsible for the substrate specificity of ubiquitin-mediated protein degradation [11].

In this review, we will discuss how protein degradation controlled by E3 ubiquitin ligases plays a fundamental role in different processes including the self-renewal, maintenance and differentiation of CSCs, highlighting its potential as effective therapeutic targets for anticancer drug development.

## 2. Cancer Stem Cells (CSCs)

**Figure 1:**
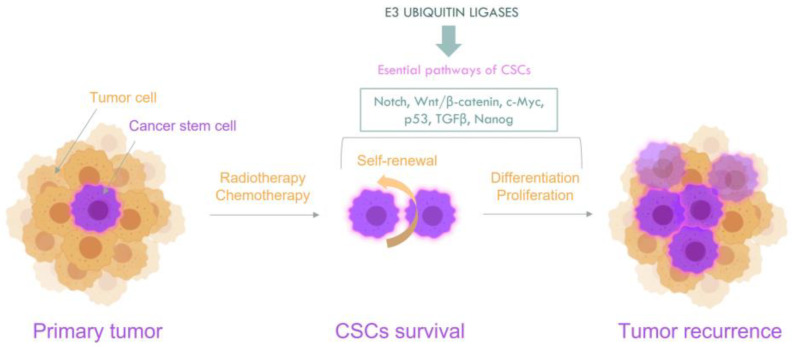
A schematic drawing of the molecular signaling pathways that influence cancer stem cell properties.

CSCs are a subpopulation of cells within the tumor that show a high capacity in self-renewal and differentiation, and are able to reconstitute tumor heterogeneity [12]. Furthermore, CSC population is also able to induce cell cycle arrest, allowing to maintain cells in a quiescent state. Given that chemotherapeutic agents commonly target the proliferating cells, CSCs in a quiescent state become resistant to chemo- and radiotherapy, a feature that is known as chemoresistance, contributing to the treatment failure and disease progression, metastasis and recurrence [13]. CSCs were first identified in leukemia and isolated through characteristic surface markers expression markers, mainly CD34^+^ and CD38^−^ [3]. However, CSCs are highly heterogeneous, and many different biomarkers have been reported in order to identify and isolate CSCs in different types of cancer including brain cancer, prostate cancer, lung cancer or melanoma [6,14,15,16,17]. Given the vast heterogeneity of CSCs, more effective biomarkers are needed. Despite the increased number of techniques that have emerged to identify CSCs, the isolation of specific CSCs is a great challenge due to their low proportion in the total tumor mass [18]. Knowing in detail the molecular profile of these cells may help provide new biomarkers and therapeutic targets. Several signaling pathways, such as Wnt/β-catenin, Notch, Hedgehog (Hh), TGF-β, JAK/STAT, PI3K/Akt and NF-κB have been shown to mediate various stem cell properties [19,20,21] (Figure 1). Moreover, CSCs are also characterized by their high plasticity, which allow them to adapt and resist to cancer treatments [22,23].

Signaling pathways that facilitate pluripotency are activated by the induction of stem cell-related transcription factors such as Oct-3/4, Sox2 and Nanog [24,25]. Indeed, stem-related transcription factors, including Oct-3/4, Sox2, Klf-4 and Nanog have been used to identify CSCs subpopulations in a variety of cancers, such colorectal cancer (CRC) [26]. On the other hand, the role of epigenetic mechanisms in the acquisition or maintenance of the CSCs phenotype has been extensively studied. Mediators of pluripotency based on epigenetic mechanisms such as DNA methylation and histone modification have been reported [27,28,29,30]. Although epigenetics plays a crucial role in CSCs function, in recent years, post-translational control has emerged as an important critical regulator [8,9]. A growing number of studies have highlighted the role of E3 ubiquitin ligases influencing the stemness of cancer cells.

## 3. Ubiquitination Process

Ubiquitination is the second most common post-translational modification with a crucial role in controlling protein degradation, interactions or activity, thus maintaining cell homeostasis [10]. During the ubiquitination process, an ubiquitin moiety, a highly conserved 76-amino acid (8.5 kDa) protein, is conjugated to a substrate protein in an ATP-dependent manner. The linking of a single ubiquitin moiety to a specific lysine residue of the target protein is named monoubiquitination. On the other hand, polyubiquitination refers to the link of two or more ubiquitin molecules to the same lysine residue of the substrate protein; and branched ubiquitination involves the link of a polyubiquitin chain to a variety of linkages instead of a single one. During the ubiquitin-dependent degradation process, three different types of enzymes participate: an E1 ubiquitin-activating enzyme, an E2 ubiquitin-conjugating enzyme and an E3 ubiquitin ligase. The E1 ubiquitin-activating enzyme activates ubiquitin in an ATP-dependent manner. Then, the activated ubiquitin is transferred to the E2 enzyme to finally be linked to a specific substrate by the action of the E3 ubiquitin ligase [31]. Ubiquitination is a reversible process, as the function of E3 ubiquitin ligases can be reverted by removing the ubiquitin molecules from the protein substrate by deubiquitinating enzymes (DUBs) [10]. The main function of this post-translational modification is degradation by proteasome, lysosome or autophagy after substrate labeling with Ub, but it also intervenes in the regulation of various intercellular signaling pathways involved in important functions such as the control of apoptosis, autophagy, the cellular cycle, transcriptional regulation and DNA repair [32,33]. Ubiquitin can also be phosphorylated, acetylated, sumoylated or neddylated, targeting the substrates for different cellular processes [34,35,36].

**Figure 2:**
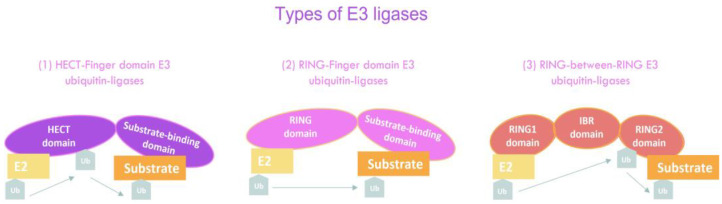
Schematic drawing of the general classification of E3 ubiquitin ligase types: (1) HECT-type E3 ubiquitin ligases bind E2 enzymes to the HECT domain and recruit the substrate with the substrate-binding domain. Ubiquitin is transferred from E2 to the HECT domain and then to the substrate protein; (2) RING-type E3 ubiquitin ligases interact with E2 enzymes to facilitate direct ubiquitin transferring to the substrate; and (3) RING-between-RING-type (RBR) E3 ubiquitin ligases bind to E2 enzymes with the RING1 domain, binds the ubiquitin with the RING2 domain and subsequently transfer the ubiquitin to the substrate.

At present, around six hundred E3 ubiquitin ligases have been reported in humans. On the contrary, only two E1 enzymes and around thirty E2s have been identified. E3 ubiquitin ligases are responsible for the recognition of substrates conferring the specificity. E3 ubiquitin ligases are classified according to the presence of different domains and the way they transfer ubiquitin to the substrate [37] (Figure 2): (1) HECT domain, the E3 enzyme transfers the ubiquitin from the E2 enzyme directly to the substrate. The HECT-type E3s are also classified into three subfamilies: NEDD4, HERC and “other HECT”. The well-known E3 ubiquitin ligases such as SMURF1 and 2, ITCH, WWP1 and 2, NEDD4 and 4-2 and HECW1 and 2 belong to the best characterized NEDD4 family; (2) RING domain, where E3 ubiquitin ligases bind to the E2 enzyme and the substrate, facilitating the ubiquitin transference. They represent the most abundant ligases with more than 500 members of the family and their role in the regulation of CSCs have been recently reviewed [38]; (3) RBR (RING-between-RING), a RING-HECT-hybrid mechanism that shares both features of RING and HECT E3 ligases. The RBR domain is composed of two RING domains (RING1 and RING2) which are separated by an in-between RING (IBR) domain. RING1 binds to the E2 conjugating enzyme and shows the same features of RING-type E3s. However, the RING2 domain behaves as an HECT domain, since it first forms a thioester bond intermediate with the ubiquitin recruited by RING1 and then transfers the ubiquitin to the substrate protein [39,40,41,42,43].

Alterations in the activity of this type of enzyme lead to the development of various serious diseases such as cancer due to the degradation of certain tumor suppressors or, on the contrary, to the lack of ubiquitination of oncogenic proteins [10]. Today, many small-molecule inhibitors are being developed to target different components of the ubiquitin proteasome system (UPS), including the proteasome, the E1 enzymes, the E2 enzymes, the E3 ligases and the DUB [10,44,45]. Importantly, the interest in E3 ubiquitin ligases as therapeutic targets is constantly increasing due to their substrate specificity, as it is expected that their inhibition causes fewer side effects [10,46]. Indeed, specific blocking the E3 ubiquitin ligase function will reduce the levels of toxicity associated with the inhibition of the proteasome, E1, E2 or ubiquitin. Related to this, different strategies targeting E3 ubiquitin ligases have been explored, including small-molecule inhibitors, microRNAs, peptides or antibodies. Although the specific inhibition of E3 ubiquitin ligase activity is a promising strategy to block cancer, a new innovative strategy came out. In this case, instead of inhibiting the E3 ligase activity, the new strategy is focused on targeted protein degradation allowing to eliminate an undruggable cancer target by using the intracellular ubiquitin-proteasome system to induce targeted protein degradation. Several ways to induce targeted protein degradation have been reported including PROTACs (proteolysis targeting chimeras), IMiDs (immunomodulatory drugs) and SNIPERs (specific and nongenetic IAP-dependent protein erasers) [47]. CSC properties are regulated by ubiquitination process [10]. Indeed, the reported proteomic analysis strongly suggests the involvement of ubiquitination in pluripotency regulation [48,49,50]. For instance, the ubiquitination of the core transcription factors, Nanog, Oct4 and Sox2, is involved in the maintenance of the stemness and pluripotency of stem cells [51,52]. Given that several contributions have recapitulated the contribution of the E3 ubiquitin ligases in cancer, epithelial-to-mesenchymal plasticity and embryonic stem cells, this issue will be not further discussed [10,42,53]. In this review, we will go in depth into the implication of the E3 ubiquitin ligases in cancer stem cells.

## 4. E3 Ubiquitin Ligases in Cancer Stem Cells

More than 80% of proteins are degraded by the ubiquitin–proteasome system. An important number of publications show that ubiquitination plays a critical role in controlling CSC properties by regulating the abundance of reported substrates related to CSCs. In recent years, several E3 ubiquitin ligases have been reported to be relevant during the CSC process, favoring the properties of pluripotent stem cells. In Table 1, the reported E3 ubiquitin ligases, their substrates and their influence on CSCs is reported.

### 4.1. RING-Finger Domain E3 Ubiquitin Ligases

#### 4.1.1. CBL Proteins

CBL proteins (Casitas B-lineage lymphoma proteins) belong to RING-type domain of the E3 ubiquitin ligases and are characterized by the presence of different domains: a N-terminal tyrosine kinase binding domain; a RING-finger domain responsible for E3 ubiquitin ligase activity; a proline-rich region; and a C-terminal ubiquitin associated domain (UBA domain) responsible for the interaction with ubiquitin [90]. This CBL family include Cbl, Cbl-b and Cbl-c, and are characterized by the recognition of the specific substrates in a phosphorylation-dependent manner [39]. Cbl and Cbl-b maintain hematopoietic stem cell properties including self-renewal and quiescence. Cbl and Cbl-b induce JAK2 ubiquitination. The depletion of these E3 ubiquitin ligases enhances JAK2 protein, therefore JAK2 signaling is activated by having an impact on cell growth in hematopoietic stem and progenitor cells. It is important to note that CBL is mutated in human leukemias accompanied by high levels of JAK2 protein and signaling [54,55]. Therefore, it is suggested that the use of JAK inhibitors may benefit patients with this type of cancer.

#### 4.1.2. SCF Family: F-Box Proteins

F-box proteins are characterized by the presence of one or more F-box domains, and are part of a SCF E3 ubiquitin ligase complex. Several F-box proteins have been linked to CSCs. For instance, FBXW7 induces the degradation of Notch, c-Myc, cyclin E and c-Jun [57,58,59], impacting on CSCs. Indeed, the depletion of FBXW7 induces stemness, EMT and metastasis in vitro and in vivo, by activating mTOR which in consequence upregulates E-cadherin transcriptional repressors such as snail, slug or snail [91]. On the other hand, FBW7 induces Notch degradation and impacts on CSCs by inducing self-renewal [92]. Indeed, the inhibition of Notch1 was reported to decrease breast CSCs and in consequence brain metastasis [93,94]. This effect of FMW7 on Noth1 was also reported in glioma, leukemia or hepatocarcinoma CSCs [58,95,96]. Importantly, Fbxw7-deficient mice show an increase in c-Myc and Notch-1 targets and it is crucial to maintain the stem cell activation [97]. Therefore, the degradation of Notch by FBW7 is a key regulator of CSCs self-renewal. FBXW7 also interacts with ZMYND8, inducing its polyubiquitination and degradation [98]. ZMYND8 is an epigenetic regulator reported as an oncogene in several tumors [99]. Reduced FBXW7 expression promotes accumulated ZMYND8 protein and enhances tumor progression and stemness in bladder cancer [98]. Another member of this large family is FBXW2, an E3 ubiquitin ligase that plays a key role in the maintenance of the property of stem cells and in the granting of drug resistance due to the binding, ubiquitination and subsequent degradation of its MSX2 substrate [56]. It is reported that the SOX2 is transcriptionally downregulated by the MSX2 [100]. Thus, a negative cascade of the FBXW2–MSX2–SOX2 axis was established, which regulates the property of stem cells and drug resistance. Indeed, under hypoxia conditions, FBXW2-mediated MSX2 ubiquitination and degradation leads to SOX2 induction via derepression. Thus, a negative cascade of the FBXW2–MSX2–SOX2 axis was reported, which regulates stem cell characteristics and drug resistance. Indeed, by the inactivation of FBXW2 using the MLN4924 inhibitor, the MSX2 accumulation was shown, and in consequence, repression of SOX2 expression leading to the suppression of stem cell property and sensitization of breast cancer cells to tamoxifen [56]. FBXW8 induces Nanog ubiquitination and degradation only when Nanog is phosphorylated at S52/71/78 [60]. Finally, FBXO11 is involved in the erythroid maturation by its action on the BAHD1 specific substrate leading to its degradation. In consequence, its transcriptional repression of important genes in erythropoiesis and mediated by PRC2 is eliminated [61]. In sum, the SCF family is implicated in stemness through the control of specific substrates involved in CSCs and they have been proposed as anticancer drug targets.

The F-box protein β-TrCP plays a dual role in the maintenance of the Wnt signaling pathway, acting on two main targets, namely ZNRF3 and β-catenin: this favors the stabilization of the former which positively regulates Wnt signaling and, on the other hand, negatively regulates Wnt signaling by targeting β-catenin [78]. Moreover, β-TrCP induces proteasomal degradation of FAP4, a basic helix–loop–helix transcription factor that was reported to control cell proliferation, stemness and EMT and is up-regulated in colorectal cancer [101]. On the other hand, the association of β-TrCP to SOX9 prevents its association to SKP1 and GLI1 substrates in pancreatic ductal adenocarcinoma (PDA). Moreover, β-TrCP is retained in the nucleus. Indeed, the deletion of β-TrCP in SOX9-deficient PDA cells restores GLI1 levels and promotes CSC properties [102].

#### 4.1.3. Ring-Finger Proteins (RNF)

This family has a wide variety of members with diverse functions, including those implicated in stemness. RNF4 contains several SUMO-interacting motifs, and a RING domain responsible for the dimerization and catalysis of ubiquitin transfer, resulting in poly-SUMO-modified substrates targeted for proteasomal degradation [103]. RNF4 targets oncoproteins such as β-catenin, c-Myc, c-Jun and Notch, inducing the ubiquitination in a phosphorylation-dependent manner. It plays a key role in CRC by regulating Wnt signaling, important for cell proliferation, maintenance of pluripotency and stem cell differentiation [71,72]. The molecule TRH 1-23 and its analogue CCW-16 were reported as a promising RNF4 inhibitor. CCW-16 is a small-molecule recruiter for RNF4, different from other reported recruiters targeting cereblon, VHL, MDM2, and cIAP. Moreover, CCW 28–3 showed higher potency for RNF4 than CCW-16. Indeed, CCW 28–3 is capable of degrading BRD4 in a RNF4-dependent manner via proteasome. The impact of these molecules in CSCs through the action on RNF4 awaits being elucidated [104]. RNF43 and its homologue ZNRF3 are defined as transmembrane E3 ubiquitin ligases. They are tumor suppressors that inhibit Wnt signaling through promoting ubiquitination and lysosomal degradation of Wnt coreceptors frizzled (FZD) and LRP6, influencing stemness. Indeed, this activity is counteracted by stem cell growth factor R-spondin [62,63,64]. RNF6 is a RING domain E3 ubiquitin ligase that induces the ubiquitination and degradation of the transducin-like enhancer of cleavage 3 (TLE3). TLE3 is a transcriptional repressor of the β-catenin/TCF4 complex. Therefore, its degradation triggers a signaling cascade that ultimately leads to the activation of the Wnt/β-catenin pathway [70]. RNF144A also influences stemness. Indeed, it has been recently reported that RNF144A induces LIN28B ubiquitination and proteasome degradation, suppressing ovarian cancer stem cells’ pluripotency and tumor progression [73].

#### 4.1.4. SIAH

SIAH1 and SIAH2 belong to the RING-domain family and are reported to induce the ubiquitination and degradation of Axin via proteasome. Moreover, when GSK3 interacts with Axin, the function of SIAH is altered, suggesting that SIAH-mediated Axin degradation may impact on the Wnt/β-catenin signaling and may favor the expression of genes related to the stem process [69].

#### 4.1.5. MDM2

The E3 ubiquitin ligase MDM2 belongs to the RING-type proteins, and its action on cancer progression has been well documented, mainly in relation to tumors of mesenchymal origin. Its best-known substrate is p53, which is polyubiquitinated and proteasome-degraded by MDM2 action. p53 induces apoptosis under different stress conditions [75]. Indeed, MDM2 mediates p53-mediated apoptosis [75]. In recent years, MDM2 has been linked to CSCs. Given that p53 regulates cell differentiation, it has been proposed that blocking the interaction between MDM2 and p53 could be a good strategy to eliminate CSCs. Indeed, MI-773, a small-molecule compound that blocks MDM2/p53 interaction increases p53 protein and its downstream target p21, in human mucoepidermoid carcinoma tissues. In consequence, the G1 cell-cycle arrest was induced, as well as cell apoptosis, in vitro. Given that patients with mucoepidermoid carcinoma express high levels of MDM2 protein, using the MI-773 inhibitor could be a good strategy to reduce CSCs by the inhibition of the MDM2–p53 interaction [76]. At present, NVP-CGM097 was selected as a small-molecule inhibitor of the protein–protein interaction between p53 and MDM2 and it is currently in phase 1 clinical development [105].

#### 4.1.6. TRIM

The tripartite motif protein family (TRIM) is in the self-renewal of CSCs. TRIM family contain a N-terminal RING finger domain, one or two B boxes domains (B1 box and B2 box), and a coil region [81]. Several members of the TRIM family have been involved in the stemness process: TRIM6 ubiquitin ligase interacts with the Myc proto-oncogene regulating its transcriptional activity during the maintenance of embryonic stem cell pluripotency, however, its potential role on CSCs awaits to be elucidated [80]. Moreover, TRIM16 induces the ubiquitination and degradation via the proteasome of Gli-1 proteins, a mediator of the hedgehog pathway [81]. TRIM19 (also named PML, promyelocytic leukemia protein) was recently linked to CSCs [82,83]. TRIM19 is highly expressed in hematopoietic stem cells. When deleting TRIM19 in leukemia-initiating cells, a reduction in survival is detected, pointing out the role of TRIM19 in leukemia-initiating cells. Moreover, an increased activity of mTOR was observed in the TRIM19−/− hematopoietic stem cells, and the mTOR inhibitor rapamycin substantially rescued the effect of TRIM19 deletion, suggesting that TRIM19 can exert its function in leukemia-initiating cells by inhibiting mTOR activity. Importantly, targeting TRIM19 eradicated quiescent cells but not all leukemia-initiating cells. On the other hand, TRIM19 was also shown to exert an essential role in hematopoietic stem cell maintenance through the regulation of PPAR signaling and fatty-acid oxidation (FAO). Indeed, the TRIM19–PPAR-δ–FAO axis controls the asymmetric division of hematopoietic stem cells and the deletion of TRIM19 and PPAR-δ as well as the inhibition of FAO which results in the symmetric commitment of hematopoietic stem cell daughter cells, further underscoring a metabolic switch for the control of hematopoietic stem cell fate with potential therapeutic implications [84]. TRIM21 was identified as an E3 ubiquitin ligase for the transcription factor Oct-1. TRIM21 enhances Oct-1 ubiquitination and degradation, reducing Oct-1 stability, crucial for the expression of the CSC ALDH1A1 marker [85]. Therefore, the authors showed that sumoylation is important for CSCs’ self-renewal and maintenance and it is proposed as target to control CSCs [85]. TRIM24 was reported to promote the stemness and invasiveness of glioblastoma cells via activating the pluripotency transcription factor Sox2 expression. The knockdown of TRIM24 reduced glioblastoma stem cell self-renewal and invasive growth. However, a specific substrate for the E3 ubiquitin ligase activity of TRIM24 awaits elucidation in order to understand the molecular mechanism by which the TRIM24–Sox2 axis may impact cancer stemness in gliobastoma [86]. TRIM28 was recently associated with breast cancer stem cells through the link to a novel long noncoding RNA named BORG. BORG expression is correlated to Nanog, Aldh1a3 and Itga6 expression, enhancing stem cell properties. BORG promotes breast cancer stem cell phenotypes through its ability to physically interact with TRIM28. Indeed, TRIM28 binds to the promoter region of Itga6, and the genetic inactivation of TRIM28 prevented BORG–TRIM28 complexes. The authors showed that BORG–TRIM28 complexes are drivers of breast CSCs phenotypes impacting on the progression of triple negative breast cancer [87]. Finally, TRIM32 was also reported to mediate the ubiquitination and subsequent degradation of c-MYC, a transcriptional regulator that controls the expression of numerous genes involved in the proliferation, growth and renewal of CSCs. Moreover, in human neuroblastoma cells, TRIM32 is recruited to the spindle poles by CDK1/cyclin B-mediated phosphorylation where it interacts with MYCN during mitosis, facilitating its proteasomal degradation, inducing asymmetric cell division and suppressing sphere formation of neuroblastoma-initiating cells [88,89].

#### 4.1.7. MARCH

Transmembrane-associated RING-CH-type finger (MARCH) proteins are a group of E3 ubiquitin ligases that represent a novel family which targets glycoproteins for lysosomal destruction. They emerged as critical regulators of immune responses. However, it remains unknown whether they play a key role in tumor development [106]. MARCH8 was recently associated with breast CSCs. Indeed, the breast cancer stem-cell marker, CD44, is a membrane protein ubiquitinated and degraded via lysosome by MARCH8. Additionally, a non-membrane protein, STAT3, was identified as another essential ubiquitinated target recognized by MARCH8. In this case, STAT3 degradation through the proteasome pathway is responsible for pro-apoptotic changes, highlighting the importance of MARCH8 as a tumor suppressor by targeting the membrane and non-membrane proteins necessary for breast cancer cell survival and metastasis. Additionally, it impairs the phenotypic functions regulated by cancer stem cells [74].

### 4.2. HECT-Domain E3 Ubiquitin Ligases

#### 4.2.1. Nedd4 Family

The NEDD4 family is the largest and best characterized family from HECT-domain E3 ubiquitin ligases [107,108]. In addition to the fact that many members have been linked to tumor progression and metastasis, only several publications have reported its role in CSCs. CD133 is a well-known marker of CSCs, recently found in extracellular vesicles. The monoubiquitination of CD133 induces cell migration and its secretion. The depletion of NEDD4 reduces CD133 ubiquitination and vesicle secretion. The lysine 848-residue at the C-terminal region is one of the sites for CD133 ubiquitination, and its mutation (K848R) does not affect CD133 degradation but reduces its secretion, which in consequence reduces cell migration [67]. NEDD4 also has an impact on intestinal stem cells. NEDD4 and NEDD4L target the intestinal stem cell marker LGR5 receptor and the central mediator of the Wnt signaling pathway DVL2 for proteasomal and lysosomal degradation. In consequence, they negatively regulate Wnt/β-catenin signaling, further underscoring the post-translational control of LGR receptors via NEDD4/NEDD4L to regulate intestinal stem cells. Indeed, the inactivation of NEDD4/NEDD4L induces Wnt activation and intestinal stem cells numbers, inducing tumor progression [68]. NEDD4 was also recently reported in CSCs as characteristic in triple-negative breast cancer (TNBC). Reduction in NEDD4 expression decreased the proliferation, migration and mammosphere formation in vitro. By proteomic analysis using breast cancer cells under the depletion of NEDD4, the alteration of CSCs markers was identified, suggesting the involvement of NEDD4 in the maintenance of CSCs in this type of tumor [109]. On the other hand, ITCH, a NEDD4-like E3 ubiquitin ligases, was reported as a regulator of large tumor suppressor 1 (LATS1) [65]. LATS1 is a serine/threonine kinase that is found to be downregulated in several types of cancer. LATS1 is an important regulator of the Hippo pathway that plays an important role in tumorigenesis, stem cell differentiation and self-renewal. Additionally, the E3 ubiquitin ligase WWP1 is also a novel negative regulator of LATS1 [66]. WWP1 promotes LATS1 polyubiquitination and degradation via the 26S proteasome pathway. The degradation of LATS1 is important for WWP1-induced increased cell proliferation in breast cancer cells, opening a novel strategy to develop drugs targeting WWP1 for suppressing breast cancer cell growth.

#### 4.2.2. Other HECT-Domain E3 Ubiquitin Ligases

The E3 ubiquitin-protein ligase E3C (UBE3C) mediates the ubiquitination and degradation of AHNAK, also known as desmoyokin. UBE3C is overexpressed in stem-like non-small cell lung cancer cells (NSCLCs), and its knockdown reduces NSCLC cancer stemness and tumorigenesis in vitro and in vivo. The overexpression of UBE3C reduces AHNAK expression. AHNAK is a cofactor of p53 acting on the inhibition of stemness-related gene transcription. The ubiquitination and degradation of AHNAK by UBE3C removes the inhibition of p53 [79]. These findings underscore the role of UBE3C as an E3 ubiquitin ligase regulator of CSCs in NSCLC. Finally, the E3 ubiquitin ligase HECTH9 (also named HUWEI, ARF-BP1 or MULE) mediates the K63-polyubiquitination of DDX17, which is a cofactor of the Drosha–DGCR8 complex in miRNA biogenesis and a transcriptional co-activator associated with cancer stem-like properties. Under hypoxia, HECTH9 controls stem-like and tumor-initiating properties. Indeed, polyubiquitinated DDX17 disassociates from the Drosha–DGCR8 complex. In consequence, the biogenesis of anti-stemness miRNAs is decreased. Moreover, an increased association of polyubiquitinated DDX17 with p300-YAP induced histone 3-lysine 56 (H3K56) acetylation leading to the transcriptional activation of stemness-related genes. These results show that the coordinated regulation of miRNA biogenesis and histone modifications through post-translational regulation by DDX17 support the stem characteristics in many cancers [77]. Moreover, HECTH9 was reported as a novel regulator of glucose metabolism. HECTH9 mediates the K63-linked ubiquitination of hexokinase 2 (HK2), regulating HK2 localization in the mitochondria and HK2-mediated glycolysis. The deficiency of HECTH9 or HK2 inhibits CSCs’ self-renewal via ROS production. These results highlight the role of the HECTH9/HK2 axis in the regulation of CSCs and CSC-associated chemoresistance [110].

## 5. Conclusions

As we reviewed in this manuscript, a growing amount of evidence highlights the role of E3 ubiquitin ligases in controlling CSCs’ characteristics, although a complete understanding of the molecular mechanism by which the ubiquitination process may impact CSCs is still needed. Many E3 ubiquitin ligases are awaiting identification to clarify the impact on self-renewal and the differentiation processes involved in CSCs. In this context, interactome or genome-wide CRSPR analyses will help address the impact of the E3 ubiquitin ligases on CSCs. Small-molecules have been reported to specifically target E3 ubiquitin ligases and have been proposed as a potential target therapy against cancer. To date, the results from preclinical and clinical studies are very promising and encouraging, particularly the PROTACs strategy which opens a new opportunity to target many undruggable proteins. Given that CSCs are a small population of cancer cells with the capacity for self-renewal, differentiation and reconstitution of tumor heterogeneity, targeting CSCs is a promising therapeutic strategy for the effective eradication of cancer [111]. In conclusion, a deep understanding of how the E3 ubiquitin ligases may influence CSCs will help develop novel therapeutic strategies against E3 ubiquitin ligases targeting CSCs to fight cancer.

## Figures and Tables

**Table 1:** E3 ubiquitin ligases involved in the regulation of cancer stem cells.

Protein	Substrates	Functional Roles	References
CBL	JAK2	Intervenes in the development of hematopoietic stem cells (HSCs).	[54,55]
FBXW2	MSX2	Involved in the pluripotency and maintenance of the properties of stem cells, through the degradation of MSX2, a repressor of SOX2.	[56]
FBXW7	Notch1, ZMYND8	Controls proteasome-mediated degradation of Notch and ZMYND8 impacting on CSCs in different types of cancers. Plays a critical role regulating the balance between self-renewal and dormancy of stem cells.	[57,58,59]
FBXW8	Nanog	Prevents the maintenance of the characteristic properties of stem cells, losing the capacity for pluripotency and self-renewal.	[60]
FBXO11	BAHD1	Targets BAHD1 influencing on the transcriptional repression mediated by PRC2 during erythropoiesis.	[61]
RNF43, ZNRF3	Frizzel and LRP6	Negative regulators of Wnt signaling by targeting its coreceptors to degradation, influencing stemness.	[62,63,64]
WWP1, ITCH	LATS1	Promote the Hippo pathway main regulator LATS1 degradation impairing stem cell differentiation and self-renewal.	[65,66]
NEDD4	LGR5, DVL2	Plays an important role for ISC self-renewal by regulating Wnt/β-catenin signaling pathway. Degrades LGR5 and DVL2, downregulating stemness and cell migration.	[67,68]
SIAH1/2	Axin	Promotes axin degradation leading to an excessive accumulation of β-catenin that favors the excessive expression of genes related to the stem process.	[69]
RNF6	TLE3	Enhances β-catenin activity by suppressing its inhibitor (TLE3). Participates in the regulation of cell proliferation and differentiation.	[70]
RNF4	β-catenin, Myc, c-Jun, Notch	Stabilizes short-lived oncogenic transcription factors. Positively regulates Wnt and Notch signaling pathways, important for pluripotency, cell proliferation and stem cell differentiation.	[71,72]
RNF144A	LIN28B	Prevents epithelial ovarian cancer (EOC) cells from acquiring stem cell properties by inducing LIN28B degradation.	[73]
MARCH8	CD44, STAT3	Degrades STAT3 and CD44 thereby impairing the phenotypic functions regulated by cancer stem cells.	[74]
MDM2	p53	Degrades one of the most important tumor suppressors (p53). Acts in multiple cellular processes, such as cell cycle regulation, DNA repair and cell differentiation.	[75,76]
HECTH9	DDX17	Promotes DDX17 poly-ubiquitination by K63 under hypoxia conditions that induces the transcription of genes related to cancer stemness properties.	[77]
β-TrCP	ZNRF3, β-catenin	Negatively regulates Wnt signaling by targeting β-catenin and positively regulates it by targeting ZNRF3.	[78]
UBE3C	AHNAK	Promotes AHNAK degradation. AHNAK is a p53 cofactor that inhibits stemness-related gene transcription. Therefore, UBEC3 acts as a key post-translational mechanism involved in maintaining the CSC properties of non-small cell lung cancer (NSCLC).	[79]
TRIM6	c-Myc	Promotes the differentiation of embryonic stem cells by enhancing the activity of central transcription factors and the induction of specific signaling pathways.	[80]
TRIM16	Gli-1	Suppresses the properties of CSCs by degrading Gli-1, the effector of the Hh signaling pathway.	[81]
TRIM19	Unknown	TRIM19 (or PML) positively regulates CSCs division and maintenance. In leukemia-initiating cells, TRIM19-null shows remarkable reduction in survival, indicating the positive role of leukemia-initiating maintenance.	[82,83,84]
TRIM21	Oct-1	Ubiquitinates Oct-1 and consequently reduces its stability, leading to a loss of self-renewal of CSCs. Oct-1 is a transcription factor that positively regulates ALDH1A1, important for the maintenance of CSC properties.	[85]
TRIM24	Sox2	Promotes stemness and invasiveness of the glioblastoma stem cells by activating the pluripotency transcription factor Sox2.	[86]
TRIM28	Unknown	Interacts with BORG and its association promotes the expression of Nanog, Aldh1a3 and Itga6 enhancing the stem cell phenotype in triple negative breast cancer.	[87]
TRIM32	c-Myc, MYCN	Promotes a RING-mediated ubiquitination and proteasomal degradation of c-Myc, inducing cell differentiation. It also induces asymmetric cell division and suppresses sphere formation in neuroblastoma initiating cells by promoting MYCN degradation.	[88,89]

## References

[1] Shackleton M., Quintana E., Fearon E.R., Morrison S.J. (2009). Heterogeneity in Cancer: Cancer Stem Cells versus Clonal Evolution. Cell.

[2] Batlle E., Clevers H. (2017). Cancer stem cells revisited. Nat. Med..

[3] Lapidot T., Sirard C., Vormoor J., Murdoch B., Hoang T., Caceres-Cortes J., Minden M., Paterson B., Caligiuri M.A., Dick J.E. (1994). A cell initiating human acute myeloid leukaemia after transplantation into SCID mice. Nature.

[4] Al-Hajj M., Wicha M.S., Benito-Hernandez A., Morrison S.J., Clarke M.F. (2003). Prospective identification of tumorigenic breast cancer cells. Proc. Natl. Acad. Sci. USA.

[5] Ricci-Vitiani L., Lombardi D.G., Pilozzi E., Biffoni M., Todaro M., Peschle C., de Maria R. (2007). Identification and expansion of human colon-cancer-initiating cells. Nature.

[6] Singh S.K., Hawkins C., Clarke I.D., Squire J.A., Bayani J., Hide T., Henkelman R.M., Cusimano M.D., Dirks P.B. (2004). Identification of human brain tumour initiating cells. Nature.

[7] O’Brien C.A., Pollett A., Gallinger S., Dick J.E. (2007). A human colon cancer cell capable of initiating tumour growth in immunodeficient mice. Nature.

[8] Avgustinova A., Benitah S.A. (2016). The epigenetics of tumour initiation: Cancer stem cells and their chromatin. Curr. Opin. Genet. Dev..

[9] Chua B.A., van der Werf I., Jamieson C., Signer R.A. (2020). Post-Transcriptional Regulation of Homeostatic, Stressed, and Malignant Stem Cells. Cell Stem Cell.

[10] Deng L., Meng T., Chen L., Wei W., Wang P. (2020). The role of ubiquitination in tumorigenesis and targeted drug discovery. Signal Transduct. Target. Ther..

[11] Weissman A.M., Shabek N., Ciechanover A. (2011). The predator becomes the prey: Regulating the ubiquitin system by ubiquitylation and degradation. Nat. Rev. Mol. Cell Biol..

[12] Pattabiraman D., Weinberg R.A. (2014). Tackling the cancer stem cells—What challenges do they pose?. Nat. Rev. Drug Discov..

[13] Visvader J.E., Lindeman G.J. (2008). Cancer stem cells in solid tumours: Accumulating evidence and unresolved questions. Nat. Cancer.

[14] Tang D.G. (2012). Understanding cancer stem cell heterogeneity and plasticity. Cell Res..

[15] Quintana E., Shackleton M., Foster H.R., Fullen D.R., Sabel M.S., Johnson T.M., Morrison S.J. (2010). Phenotypic Heterogeneity among Tumorigenic Melanoma Cells from Patients that Is Reversible and Not Hierarchically Organized. Cancer Cell.

[16] van den Hoogen C., van der Horst G., Cheung H., Buijs J.T., Lippitt J.M., Guzmán-Ramírez N., Hamdy F.C., Eaton C.L., Thalmann G.N., Cecchini M.G. (2010). High aldehyde dehydrogenase activity identifies tumor-initiating and metastasis-initiating cells in human prostate cancer. Cancer Res..

[17] Zhang W.C., Shyh-Chang N., Yang H., Rai A., Umashankar S., Ma S., Soh B.S., Sun L.L., Tai B.C., Nga M.E. (2012). Glycine Decarboxylase Activity Drives Non-Small Cell Lung Cancer Tumor-Initiating Cells and Tumorigenesis. Cell.

[18] Dobbin Z.C., Landen C.N. (2013). Isolation and Characterization of Potential Cancer Stem Cells from Solid Human Tumors—Potential Applications. Curr. Protoc. Pharmacol..

[19] Kanwar S.S., Yu Y., Nautiyal J., Patel B.B., Majumdar A.P. (2010). The Wnt/β-catenin pathway regulates growth and maintenance of colonospheres. Mol. Cancer.

[20] Pardal R., Clarke M.F., Morrison S.J. (2003). Applying the principles of stem-cell biology to cancer. Nat. Rev. Cancer.

[21] Wang J., Wakeman T.P., Lathia J.D., Hjelmeland A.B., Wang X.F., White R.R., Rich J.N., Sullenger B.A. (2010). Notch promotes radioresistance of glioma stem cells. Stem Cells.

[22] Sarrió D., Franklin C.K., Mackay A., Reis-Filho J.S., Isacke C.M. (2012). Epithelial and Mesenchymal Subpopulations within Normal Basal Breast Cell Lines Exhibit Distinct Stem Cell/Progenitor Properties. Stem Cells.

[23] De Las Rivas J., Brozovic A., Izraely S., Casas-Pais A., Witz I.P., Figueroa A. (2021). Cancer drug resistance induced by EMT: Novel therapeutic strategies. Arch. Toxicol..

[24] Hadjimichael C., Chanoumidou K., Papadopoulou N., Arampatzi P., Papamatheakis J., Kretsovali A. (2015). Common stemness regulators of embryonic and cancer stem cells. World J. Stem Cells.

[25] Takahashi K., Yamanaka S. (2006). Induction of Pluripotent Stem Cells from Mouse Embryonic and Adult Fibroblast Cultures by Defined Factors. Cell.

[26] Pádua D., Figueira P., Ribeiro I., Almeida R., Mesquita P. (2020). The Relevance of Transcription Factors in Gastric and Colorectal Cancer Stem Cells Identification and Eradication. Front. Cell Dev. Biol..

[27] Kreso A., O’Brien C.A., van Galen P., Gan O.I., Notta F., Brown A.M.K., Ng K., Ma J., Wienholds E., Dunant C. (2013). Variable Clonal Repopulation Dynamics Influence Chemotherapy Response in Colorectal Cancer. Science.

[28] Saygin C., Matei D., Majeti R., Reizes O., Lathia J.D. (2019). Targeting Cancer Stemness in the Clinic: From Hype to Hope. Cell Stem Cell.

[29] Berdasco M., Esteller M. (2011). DNA methylation in stem cell renewal and multipotency. Stem Cell Res. Ther..

[30] Muñoz P., Iliou M.S., Esteller M. (2012). Epigenetic alterations involved in cancer stem cell reprogramming. Mol. Oncol..

[31] Hershko A., Ciechanover A., Varshavsky A. (2000). Basic Medical Research Award. The ubiquitin system. Nat. Med..

[32] Popovic D., Vucic D., Dikic I. (2014). Ubiquitination in disease pathogenesis and treatment. Nat. Med..

[33] Rape M. (2018). Ubiquitylation at the crossroads of development and disease. Nat. Rev. Mol. Cell Biol..

[34] Galisson F., Mahrouche L., Courcelles M., Bonneil E., Meloche S., Chelbi-Alix M.K., Thibault P. (2011). A Novel Proteomics Approach to Identify SUMOylated Proteins and Their Modification Sites in Human Cells. Mol. Cell. Proteom..

[35] Ohh M., Kim W.Y., Moslehi J.J., Chen Y., Chau V., Read M.A., Kaelin W.G. (2002). An intact NEDD8 pathway is required for Cullin-dependent ubiquitylation in mammalian cells. EMBO Rep..

[36] Swaney D.L., Beltrao P., Starita L., Guo A., Rush J., Fields S., Krogan N.J., Villén J. (2013). Global analysis of phosphorylation and ubiquitylation cross-talk in protein degradation. Nat. Methods.

[37] Buetow L., Huang D.T. (2016). Structural insights into the catalysis and regulation of E3 ubiquitin ligases. Nat. Rev. Mol. Cell Biol..

[38] Kang B., Sun X.-H. (2014). Regulation of cancer stem cells by RING finger ubiquitin ligases. Stem Cell Investig..

[39] Cooper J.A., Kaneko T., Li S.S.C. (2015). Cell Regulation by Phosphotyrosine-Targeted Ubiquitin Ligases. Mol. Cell. Biol..

[40] Berndsen C., Wolberger C. (2014). New insights into ubiquitin E3 ligase mechanism. Nat. Struct. Mol. Biol..

[41] Scheffner M., Kumar S. (2014). Mammalian HECT ubiquitin-protein ligases: Biological and pathophysiological aspects. Biochim. Biophys. Acta.

[42] Rodríguez-Alonso A., Casas-Pais A., Roca-Lema D., Graña B., Romay G., Figueroa A. (2020). Regulation of Epithelial–Mesenchymal Plasticity by the E3 Ubiquitin-Ligases in Cancer. Cancers.

[43] Uchida C., Kitagawa M. (2016). RING-, HECT-, and RBR-type E3 Ubiquitin Ligases: Involvement in Human Cancer. Curr. Cancer Drug Targets.

[44] Tian M., Zeng T., Liu M., Han S., Lin H., Lin Q., Li L., Jiang T., Li G., Lin H. (2019). A cell-based high-throughput screening method based on a ubiquitin-reference technique for identifying modulators of E3 ligases. J. Biol. Chem..

[45] Appel A. (2011). Drugs: More shots on target. Nature.

[46] Huang X., Dixit V.M. (2016). Drugging the undruggables: Exploring the ubiquitin system for drug development. Cell Res..

[47] Schapira M., Calabrese M.F., Bullock A.N., Crews C.M. (2019). Targeted protein degradation: Expanding the toolbox. Nat. Rev. Drug Discov..

[48] Buckley S.M., Aranda-Orgilles B., Strikoudis A., Apostolou E., Loizou E., Moran-Crusio K., Farnsworth C.L., Koller A.A., Dasgupta R., Silva J.C. (2012). Regulation of Pluripotency and Cellular Reprogramming by the Ubiquitin-Proteasome System. Cell Stem Cell.

[49] Baharvand H., Hajheidari M., Ashtiani S.K., Salekdeh G.H. (2006). Proteomic signature of human embryonic stem cells. Proteomics.

[50] Vilchez D., Boyer L., Morantte I., Lutz M., Merkwirth C., Joyce D., Spencer B., Page L., Masliah E., Berggren W.T. (2012). Increased proteasome activity in human embryonic stem cells is regulated by PSMD11. Nature.

[51] Cai N., Li M., Qu J., Liu G.-H., Belmonte J.C.I. (2012). Post-translational modulation of pluripotency. J. Mol. Cell Biol..

[52] Suresh B., Lee J., Kim K.-S., Ramakrishna S. (2016). The Importance of Ubiquitination and Deubiquitination in Cellular Reprogramming. Stem Cells Int..

[53] Strikoudis A., Guillamot M., Aifantis I. (2014). Regulation of stem cell function by protein ubiquitylation. EMBO Rep..

[54] Lv K., Jiang J., Donaghy R., Riling C.R., Cheng Y., Chandra V., Rozenova K., An W., Mohapatra B.C., Goetz B.T. (2017). CBL family E3 ubiquitin ligases control JAK2 ubiquitination and stability in hematopoietic stem cells and myeloid malignancies. Genes Dev..

[55] An W., Nadeau S.A., Mohapatra B., Feng D., Zutshi N., Storck M., Arya P., Talmadge J.E., Meza J.L., Band V. (2015). Loss of Cbl and Cbl-b ubiquitin ligases abrogates hematopoietic stem cell quiescence and sensitizes leukemic disease to chemotherapy. Oncotarget.

[56] Yin Y., Xie C.-M., Li H., Tan M., Chen G., Schiff R., Xiong X., Sun Y. (2019). The FBXW2–MSX2–SOX2 axis regulates stem cell property and drug resistance of cancer cells. Proc. Natl. Acad. Sci. USA.

[57] Gallo L.H., Ko J., Donoghue D.J. (2017). The importance of regulatory ubiquitination in cancer and metastasis. Cell Cycle.

[58] Wang Z., Inuzuka H., Fukushima H., Wan L., Gao D., Shaik S., Sarkar F.H., Wei W. (2011). Emerging roles of the FBW7 tumour suppressor in stem cell differentiation. EMBO Rep..

[59] Yeh C.-H., Bellon M., Nicot C. (2018). FBXW7: A critical tumor suppressor of human cancers. Mol. Cancer.

[60] Kim S.-H., Kim M.O., Cho Y.-Y., Yao K., Kim D.J., Jeong C.-H., Yu D.H., Bae K.B., Cho E.J., Jung S.K. (2014). ERK1 phosphorylates Nanog to regulate protein stability and stem cell self-renewal. Stem Cell Res..

[61] Xu P., Scott D.C., Xu B., Yao Y., Feng R., Cheng L., Mayberry K., Wang Y.-D., Bi W., Palmer L.E. (2021). FBXO11-mediated proteolysis of BAHD1 relieves PRC2-dependent transcriptional repression in erythropoiesis. Blood.

[62] Hao H.-X., Xie Y., Zhang Y., Charlat O., Oster E., Avello M., Lei H., Mickanin C., Liu D., Ruffner H. (2012). ZNRF3 promotes Wnt receptor turnover in an R-spondin-sensitive manner. Nature.

[63] Koo B.-K., Spit M., Jordens I., Low T.Y., Stange D., Van De Wetering M., Van Es J.H., Mohammed S., Heck A., Maurice M. (2012). Tumour suppressor RNF43 is a stem-cell E3 ligase that induces endocytosis of Wnt receptors. Nature.

[64] Lebensohn A.M., Rohatgi R. (2018). R-spondins can potentiate WNT signaling without LGRs. Elife.

[65] Ho K.C., Zhou Z., She Y.-M., Chun A., Cyr T.D., Yang X. (2011). Itch E3 ubiquitin ligase regulates large tumor suppressor 1 stability. Proc. Natl. Acad. Sci. USA.

[66] Yeung B., Ho K.-C., Yang X. (2013). WWP1 E3 Ligase Targets LATS1 for Ubiquitin-Mediated Degradation in Breast Cancer Cells. PLoS ONE.

[67] Yang F., Xing Y., Li Y., Chen X., Jiang J., Ai Z., Wei Y. (2018). Monoubiquitination of Cancer Stem Cell Marker CD133 at Lysine 848 Regulates Its Secretion and Promotes Cell Migration. Mol. Cell. Biol..

[68] Novellasdemunt L., Kucharska A., Jamieson C., Prange-Barczynska M., Baulies A., Antas P., van der Vaart J., Gehart H., Maurice M.M., Li V.S. (2020). NEDD4 and NEDD4L regulate Wnt signalling and intestinal stem cell priming by degrading LGR5 receptor. EMBO J..

[69] Ji L., Jiang B., Jiang X., Charlat O., Chen A., Mickanin C., Bauer A., Xu W., Yan X., Cong F. (2017). The SIAH E3 ubiquitin ligases promote Wnt/β-catenin signaling through mediating Wnt-induced Axin degradation. Genes Dev..

[70] Liu L., Zhang Y., Wong C.C., Zhang J., Dong Y., Li X., Kang W., Chan F.K., Sung J.J.Y., Yu J. (2018). RNF6 Promotes Colorectal Cancer by Activating the Wnt/β-Catenin Pathway via Ubiquitination of TLE3. Cancer Res..

[71] Thomas J.J., Abed M., Heuberger J., Novak R., Zohar Y., Lopez A.P.B., Trausch-Azar J.S., Ilagan M.X., Benhamou D., Dittmar G. (2016). RNF4-Dependent Oncogene Activation by Protein Stabilization. Cell Rep..

[72] Liu L., Wong C.C., Gong B., Yu J. (2018). Functional significance and therapeutic implication of ring-type E3 ligases in colorectal cancer. Oncogene.

[73] Li Y., Wang J., Wang F., Chen W., Gao C., Wang J. (2021). RNF144A suppresses ovarian cancer stem cell properties and tumor progression through regulation of LIN28B degradation via the ubiquitin-proteasome pathway. Cell Biol. Toxicol..

[74] Chen W., Patel D., Jia Y., Yu Z., Liu X., Shi H., Liu H. (2021). MARCH8 Suppresses Tumor Metastasis and Mediates Degradation of STAT3 and CD44 in Breast Cancer Cells. Cancers.

[75] Chène P. (2003). Inhibiting the p53–MDM2 interaction: An important target for cancer therapy. Nat. Cancer.

[76] Andrews A., Warner K., Rodriguez-Ramirez C., Pearson A.T., Nör F., Zhang Z., Kerk S., Kulkarni A.S., Helman J.I., Brenner J.C. (2019). Ablation of Cancer Stem Cells by Therapeutic Inhibition of the MDM2–p53 Interaction in Mucoepidermoid Carcinoma. Clin. Cancer Res..

[77] Kao S.-H., Cheng W.-C., Wang Y.-T., Wu H.-T., Yeh H.-Y., Chen Y.-J., Tsai M.-H., Wu K.-J. (2019). Regulation of miRNA biogenesis and histone modification by K63-polyubiquitinated DDX17 controls cancer stem-like features. Cancer Res..

[78] Ci Y., Li X., Chen M., Zhong J., North B.J., Inuzuka H., He X., Li Y., Guo J., Dai X. (2018). SCFβ-TRCP E3 ubiquitin ligase targets the tumor suppressor ZNRF3 for ubiquitination and degradation. Protein Cell.

[79] Gu J., Mao W., Ren W., Xu F., Zhu Q., Lu C., Lin Z., Zhang Z., Chu Y., Liu R. (2019). Ubiquitin-protein ligase E3C maintains non-small-cell lung cancer stemness by targeting AHNAK-p53 complex. Cancer Lett..

[80] Sato T., Okumura F., Ariga T., Hatakeyama S. (2012). TRIM6 interacts with c-Myc and maintains pluripotency of mouse embryonal stem cells. J. Cell Sci..

[81] Jaworska A.M., Wlodarczyk N.A., Mackiewicz A., Czerwinska P. (2019). The role of TRIM family proteins in the regulation of cancer stem cell self-renewal. Stem Cells.

[82] Ito K., Bernardi R., Morotti A., Matsuoka S., Saglio G., Ikeda Y., Rosenblatt J., Avigan D.E., Teruya-Feldstein J., Pandolfi P.P. (2008). PML targeting eradicates quiescent leukaemia-initiating cells. Nature.

[83] Zhou W., Bao S. (2014). PML-mediated signaling and its role in cancer stem cells. Oncogene.

[84] Ito K., Carracedo A., Weiss D., Arai F., Ala U., Avigan D.E., Schafer Z.T., Evans R.M., Suda T., Lee C.-H. (2012). A PML–PPAR-δ pathway for fatty acid oxidation regulates hematopoietic stem cell maintenance. Nat. Med..

[85] Du L., Li Y.-J., Fakih M., Wiatrek R.L., Duldulao M., Chen Z., Chu P., Garcia-Aguilar J., Chen Y. (2016). Role of SUMO activating enzyme in cancer stem cell maintenance and self-renewal. Nat. Commun..

[86] Zhang L.-H., Yin Y.-H., Chen H.-Z., Feng S.-Y., Liu J.-L., Chen L., Fu W.-L., Sun G.-C., Yu X.-G., Xu D.-G. (2020). TRIM24 promotes stemness and invasiveness of glioblastoma cells via activating Sox2 expression. Neuro-Oncology.

[87] Parker K.A., Gooding A.J., Valadkhan S., Schiemann W.P. (2021). lncRNA BORG:TRIM28 Complexes Drive Metastatic Progression by Inducing α6 Integrin/CD49f Expression in Breast Cancer Stem Cells. Mol. Cancer Res..

[88] Izumi H., Kaneko Y. (2014). Trim32 Facilitates Degradation of MYCN on Spindle Poles and Induces Asymmetric Cell Division in Human Neuroblastoma Cells. Cancer Res..

[89] Izumi H., Kaneko Y. (2014). Symmetry breaking in human neuroblastoma cells. Mol. Cell. Oncol..

[90] Liyasova M.S., Ma K., Lipkowitz S. (2015). Molecular Pathways: Cbl Proteins in Tumorigenesis and Antitumor Immunity—Opportunities for Cancer Treatment. Clin. Cancer Res..

[91] Yang H., Lu X., Liu Z., Chen L., Xu Y., Wang Y., Wei G., Chen Y. (2015). FBXW7 suppresses epithelial-mesenchymal transition, stemness and metastatic potential of cholangiocarcinoma cells. Oncotarget.

[92] Ranganathan P., Weaver K.L., Capobianco A.J. (2011). Notch signalling in solid tumours: A little bit of everything but not all the time. Nat. Rev. Cancer.

[93] Harrison H., Farnie G., Brennan K.R., Clarke R. (2010). Breast Cancer Stem Cells: Something Out of Notching?. Cancer Res..

[94] McGowan P.M., Simedrea C., Ribot E.J., Foster P.J., Palmieri D., Steeg P.S., Allan A.L., Chambers A.F. (2011). Notch1 Inhibition Alters the CD44hi/CD24lo Population and Reduces the Formation of Brain Metastases from Breast Cancer. Mol. Cancer Res..

[95] Nishina S.-I., Shiraha H., Nakanishi Y., Tanaka S., Matsubara M., Takaoka N., Uemura M., Horiguchi S., Kataoka J., Iwamuro M. (2011). Restored expression of the tumor suppressor gene RUNX3 reduces cancer stem cells in hepatocellular carcinoma by suppressing Jagged1-Notch signaling. Oncol. Rep..

[96] Alcalay M., Meani N., Gelmetti V., Fantozzi A., Fagioli M., Orleth A., Riganelli D., Sebastiani C., Cappelli E., Casciari C. (2003). Acute myeloid leukemia fusion proteins deregulate genes involved in stem cell maintenance and DNA repair. J. Clin. Investig..

[97] Matsuoka S., Oike Y., Onoyama I., Iwama A., Arai F., Takubo K., Mashimo Y., Oguro H., Nitta E., Ito K. (2008). Fbxw7 acts as a critical fail-safe against premature loss of hematopoietic stem cells and development of T-ALL. Genes Dev..

[98] Qiu F., Jin Y., Pu J., Huang Y., Hou J., Zhao X., Lu Y. (2021). Aberrant FBXW7-mediated ubiquitination and degradation of ZMYND8 enhances tumor progression and stemness in bladder cancer. Exp. Cell Res..

[99] Chen Y., Tsai Y.-H., Tseng S.-H. (2021). Regulation of ZMYND8 to Treat Cancer. Molecules.

[100] Wu Q., Zhang L., Su P., Lei X., Liu X., Wang H., Lu L., Bai Y., Xiong T., Li D. (2015). MSX2 mediates entry of human pluripotent stem cells into mesendoderm by simultaneously suppressing SOX2 and activating NODAL signaling. Cell Res..

[101] D’Annibale S., Kim J., Magliozzi R., Low T.Y., Mohammed S., Heck A., Guardavaccaro D. (2014). Proteasome-dependent Degradation of Transcription Factor Activating Enhancer-binding Protein 4 (TFAP4) Controls Mitotic Division. J. Biol. Chem..

[102] Deng W., Vanderbilt D.B., Lin C.-C., Martin K.H., Brundage K.M., Ruppert J.M. (2015). SOX9 inhibits β-TrCP-mediated protein degradation to promote nuclear GLI1 expression and cancer stem cell properties. J. Cell Sci..

[103] Plechanovová A., Jaffray E.G., McMahon S.A., Johnson K.A., Navrátilová I., Naismith J.H., Hay R.T. (2011). Mechanism of ubiquitylation by dimeric RING ligase RNF4. Nat. Struct. Mol. Biol..

[104] Ward C.C., Kleinman J.I., Brittain S.M., Lee P.S., Chung C.Y.S., Kim K., Petri Y., Thomas J.R., Tallarico J.A., McKenna J.M. (2019). Covalent Ligand Screening Uncovers a RNF4 E3 Ligase Recruiter for Targeted Protein Degradation Applications. ACS Chem. Biol..

[105] Holzer P., Masuya K., Furet P., Kallen J., Valat-Stachyra T., Ferretti S., Berghausen J., Bouisset-Leonard M., Buschmann N., Pissot-Soldermann C. (2015). Discovery of a Dihydroisoquinolinone Derivative (NVP-CGM097): A Highly Potent and Selective MDM2 Inhibitor Undergoing Phase 1 Clinical Trials in p53wt Tumors. J. Med. Chem..

[106] Wang X., Herr R.A., Hansen T. (2008). Viral and cellular MARCH ubiquitin ligases and cancer. Semin. Cancer Biol..

[107] Eaton D.C., Malik B., Bao H.-F., Yu L., Jain L. (2010). Regulation of Epithelial Sodium Channel Trafficking by Ubiquitination. Proc. Am. Thorac. Soc..

[108] Wang Z., Liu Z., Chen X., Li J., Yao W., Huang S., Gu A., Lei Q.-Y., Mao Y., Wen W. (2019). A multi-lock inhibitory mechanism for fine-tuning enzyme activities of the HECT family E3 ligases. Nat. Commun..

[109] Jeon S.-A., Kim D.W., Lee D.-B., Cho J.-Y. (2020). NEDD4 Plays Roles in the Maintenance of Breast Cancer Stem Cell Characteristics. Front. Oncol..

[110] Lee H.-J., Li C.-F., Ruan D., He J., Montal E.D., Lorenz S., Girnun G.D., Chan C.-H. (2019). Non-proteolytic ubiquitination of Hexokinase 2 by HectH9 controls tumor metabolism and cancer stem cell expansion. Nat. Commun..

[111] Shibata M., Hoque M.O. (2019). Targeting Cancer Stem Cells: A Strategy for Effective Eradication of Cancer. Cancers.

